# Mesenchymal Tumors of the Breast: Fibroblastic/Myofibroblastic Lesions and Other Lesions

**DOI:** 10.3390/curroncol30050338

**Published:** 2023-04-24

**Authors:** Riordan Azam, Miralem Mrkonjic, Abha Gupta, Rebecca Gladdy, Andrea M. Covelli

**Affiliations:** 1 Postgraduate Medical Education, University of Toronto, Toronto, ON M5G 1V7, Canada; 2 Department of Laboratory Medicine and Pathobiology, University of Toronto, Toronto, ON M5G 1X5, Canada; 3 Mount Sinai Hospital and Princess Margaret Cancer Center, University of Toronto, Toronto, ON M5G 2C4, Canada; 4 Department of Medical Oncology, University of Toronto, Toronto, ON M5G 1X8, Canada; 5 The Hospital for Sick Children and Princess Margaret Cancer Center, University of Toronto, Toronto, ON M5G 1X5, Canada; 6 Division of Surgical Oncology, Department of Surgery, University of Toronto, Toronto, ON M5G 1X5, Canada

**Keywords:** mesenchymal tumors, spindle cell tumors, fibroblastic, myofibroblastic, breast tumors

## Abstract

Mesenchymal breast tumors are a rare and diverse group of tumors that present some of the most challenging cases for multidisciplinary breast cancer teams. As a result of overlapping morphologies and a lack of large-scale studies on these tumors, practices are often heterogeneous and slow to evolve. Herein, we present a non-systematic review that focuses on progress, or lack thereof, in the field of mesenchymal breast tumors. We focus on tumors originating from fibroblastic/myofibroblastic cells and tumors originating from less common cellular origins (smooth muscle, neural tissue, adipose tissue, vascular tissue, etc.).

## 1. Introduction

Mesenchymal breast tumors are a rare and diverse group of tumors that present some of the most challenging cases for multidisciplinary breast cancer teams. Upfront diagnosis is complicated by the overlapping morphologies of benign and malignant lesions from different cell lineages. The diagnostic landscape has changed and has recently been facilitated by the growing use and understanding of molecular testing. Management is equally complex as tumor rarity (except for fibroadenomas) prevents large-scale studies that would otherwise facilitate a thorough disease comprehension and an evidence-based practice. As a result, practices can be heterogeneous. As international interconnectivity increases and multicenter collaboratives for tumor sample analyses are established, such as the Rare Tumor Initiative started by MD Anderson in 2019, knowledge and practice are changing [1]. 

### Objectives and Methods

**Figure 1:**
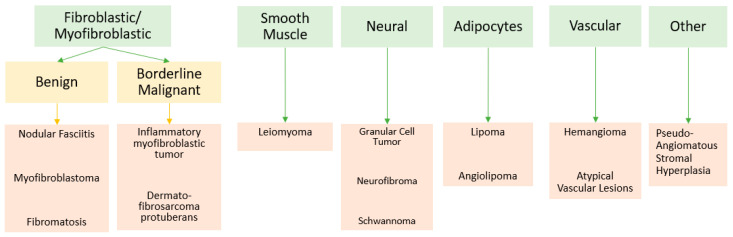
Mesenchymal Tumors of the Breast, Fibroblastic/Myofibroblastic Origin, and Tumors of “Other” Cellular Origin.

Presented below is a non-systematic review of mesenchymal breast tumors categorized by cell lineage (see Figure 1). The focus herein is on mesenchymal tumors originating from fibroblastic/myofibroblastic cells as well as other more unusual cellular origins including smooth muscle, neural tissue, adipose tissue, vascular tissue, etc. 

The objective is to update the existing literature on rare tumor types and to act as a reference paper for treating physicians. We focus on progress, or lack thereof, in the landscape of their diagnosis and management over the past 20 years. All pictures and figures in this review were originally created ot extracted from pathology records at Mount Sinai Hospital in Toronto, Canada, and had no identifying information and no requirement for Research Ethics Board approval. 

## 2. Fibroblastic/Myofibroblastic Lesions

### 2.1. Nodular Fasciitis

Nodular fasciitis was first described as “subcutaneous pseudosarcomatous fibromatosis” by Konwaler et al. in 1955 [2,3]. It is an uncommon, benign fibroblastic lesion occurring in young patients on the upper extremities, head, neck [4], and, occasionally, breasts. Extra-mammary NF is classified based on origin as subcutaneous, intra-muscular, fascial, etc. Most mammary cases likely originate from the subcutis and secondarily involve breast parenchyma [5]. The scarcity of cases contributes to 60% of cases being misclassified and 20–30% being over-diagnosed [5,6]. 

NF presents as a painless mass; its rapid growth can differentiate it from other benign tumors [4,6]. It has been described as a reactive lesion, though a history of trauma is only present in 10–15% [7]. Radiographically, these lesions closely resemble malignancies. On ultrasound (US), they are hypoechoic with a spiculated appearance [7]. There may be some hyperechoicity around the lesions that Lee et al., while evaluating musculoskeletal nodular fasciitis, hypothesized is related to infiltrating inflammatory changes or septal fibrosis [8]. Similarly, mammographically, these lesions appear hyperdense with indistinct margins [9]. 

**Figure 2:**
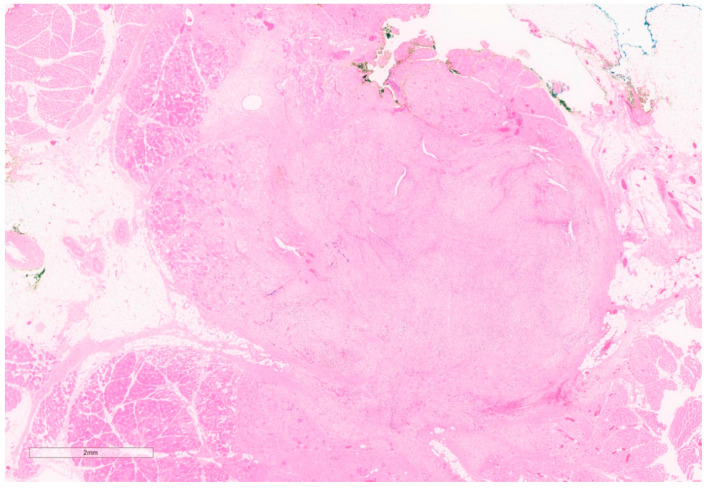
Nodular Fasciitis, low-power field, well-encapsulated mass.

**Figure 3:**
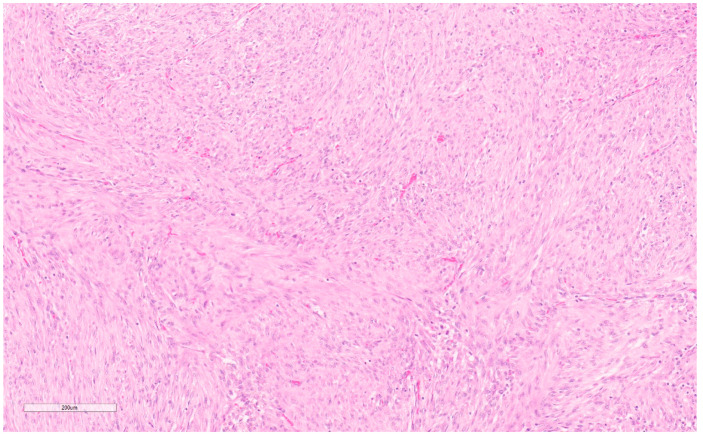
Nodular fasciitis, high power field (hpf), fibroblasts, and myofibroblasts with occasional lymphocytes and extravasated RBCs.

Well-sampled core needle biopsies (CNBs), with classic features, radio-pathologic concordance, and review by an expert pathologist may be sufficient for diagnosis [5]. However, usually, CNBs and Fine Needle Aspirations (FNAs) are insufficient. An excisional biopsy is almost always required and recommended, for definitive diagnosis [4], especially in the elderly [10,11] given the similarity to spindle cell carcinomas and sarcomas [11]. Grossly, after excision they are well encapsulated masses (see Figure 2). Microscopic key features include clusters of fibroblasts in short bundles with myxoid stroma accompanied by inflammatory cells (predominantly lymphocytes) and extravasated blood (see Figure 3) [12]. These features have been classified into three subtypes, namely, myxoid, cellular, and fibrous, which are roughly correlated to the duration of the lesion with older lesions being more fibrous and younger lesions, being more myxoid [7]. Though mitotic figures are often present, the lack of atypical mitoses differentiates them from sarcomas and carcinomas [4,5,6]. 

On immunohistochemistry (IHC), NF stains are positive for Smooth Muscle Actin (SMA) [4]. Though sensitive, it is not specific [5], and ruling out metaplastic carcinomas requires a panel of low and high-molecular-weight keratins and p63 stains. In 2011, NF was noted to be associated with a fusion gene Ubiquitin Specific Protease 6 (USP6) [4] most often with Myosin Heavy Chain 9 (MYH9) [4]. This gene rearrangement can support the diagnosis in poorly sampled cases [5]; especially given that with a cutoff of 9–10% on Fluorescent In Situ Hybridization (FISH), it carries a sensitivity of 93% and a specificity of 100% (in any location) [13]. This clonal proliferation defies the theory that these lesions are reactive [13] and suggests that NF may lie along a biologic spectrum with other USP6 gene-disordered lesions (such as aneurysmal bone cysts and myositis ossificans) that have similar clinical behaviors [14]. 

Excision is also commonly recommended for therapeutic purposes, citing diagnostic difficulties and lack of data, and known potential for recurrence. Recurrences have been reported very infrequently in mammary cases. There is limited research on healthy tissue margins as an influential factor for recurrence [4,6]. One review demonstrated spontaneous resolution without resection within 1 month to 2 years in 4 of their 20 patients [6] with other reports noting the same [15]. Extra-mammary NF has shown promising results with intra-lesional corticosteroids as far back as 1999 [16]. A 2015 series by Ho Oh on facial nodular fasciitis demonstrated 7 post-excision recurrences in 16 patients [17]. Intra-lesional triamcinolone (ILTA) resulted in the resolution of all seven recurrences and five lesions primarily treated with ILTA [17]. No metastases are noted in the literature. 

#### Summary of Updates

Advancements in genetic testing and staining abilities have made NF easier to identify on pre-operative biopsy samples. In the setting of radio-pathologic concordance, the increasing international literature and the number of cases have demonstrated a lack of metastases and malignant transformation supporting the option of conservative management. There has been a note of spontaneous resolutions and promising results of ILTA.

### 2.2. Myofibroblastoma

Mammary myofibroblastomas were initially described less than 40 years ago, by Wargotz in 1987 [18] in a series of 16 patients. First identified in the breast, other anatomic distributions have been described since 2001 [19,20]. They are myofibroblastic in origin with a potentially reactive component [18].

Myofibroblasts often respond to injury, producing transforming growth factor beta which stimulates the production of smooth muscle actin fibers. In myofibroblastomas, this process that the wound normally accelerates wound healing through wound contraction is excessive and disproportionate [21].

Myofibroblastomas have a supposed male predominance [18,21] with an association with gynaecomastia, though others have refuted the gender predilection [22]. They appear as slow-growing, solid, and mobile masses. 

They are non-specific about imaging. On mammography and US, they are well-circumscribed with occasional coarse calcifications on the former and with homogeneity and hypoechogenicity on the latter [21]. On MRI, they appear hyperintense on T2 and are homogenous, circumscribed with internal septations [21]. Recently, in tumors of epithelial origin, the Apparent Diffusion Coefficient (ADC) has been found to be lower in malignant differentials than benign ones [23]. Consistent with this, one case report demonstrated myofibroblastomas to have low ADCs [21,24]. 

Generally, for a complete triple assessment, a CNB is required rather than an FNA. FNA will generally only show whorls of spindle cells that are non-specific [22]. 

**Figure 4:**
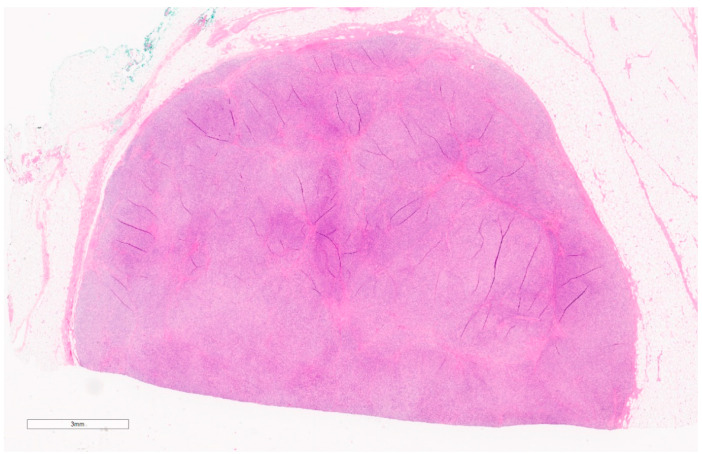
Myofibroblastoma, low power field, well-encapsulated mass.

**Figure 5:**
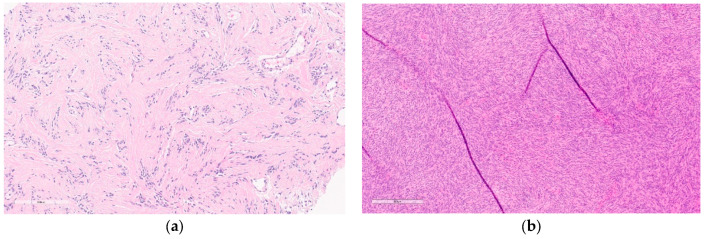
Myofibroblastoma, hpf, (**a**) haphazard fascicles with variably hyalinized collagen (**b**) storiform architecture.

Macroscopically, they are yellow/tan well-circumscribed tumors (see Figure 4) [20]. Microscopically, they appear as random arrangements of fascicular bipolar bland spindle cells and interspersed adipocytes in the collagenous and myxoid background surrounded by a pseudo-capsule (see Figure 5) [21]. Mitotic figures and necrosis are generally absent. There are a variety of histologic patterns including collagenous, cellular, lipomatous, infiltrative, leiomyomatous, epithelioid, myxoid, and deciduoid. IHC stains for desmin and CD34 tested positive in 89% and 91%, respectively, in Howitt’s series [20,21] of 143 patients with mammary and extra-mammary myofibroblastomas. Myofibroblastomas may also be positive for CD10, CD99, estrogen, and progesterone receptors (ER and PR) and focally positive for H-caldesmon (in leiomyomatous variants) [25]. S-100 markers, HMB45, epithelial markers (EMA and pancytokeratins), and c-kit (CD 117) are consistently negative [25]. 

In the largest published series of 143 cases of mammary myofibroblastoma, Retinoblastoma (RB) analysis was negative in 92% [20]. They have been considered to belong to a 13q/RB group of tumors involving the loss of Rb expression through a deletion at chromosome 13q14. This group includes two similar tumors: spindle cell lipomas and cellular angiofibromas which are differentiated from myofibroblastomas by the lack of desmin and the lack of adipocytes, respectively. This group’s shared genetics, similar morphology, and similar clinical behavior (with the exception of aneurysmal bone tumors) have implied a potential spectrum of lesions rather than separate entities [20]. 

Since their discovery, MFs have been managed by local excision. There were no identified case reports of observation for this benign lesion; their natural history without local excision is therefore unclear [18,21]). Some have been managed by mastectomy, due to size or misdiagnosis as sarcomas [22]. Howitt reports two late (>10 years) local recurrences. Though the anatomic location of these was not mentioned, breast MF may carry the potential to recur [20].

#### Summary of Updates

Myofibroblastomas’ genetic profile has evolved over the last 20 years with the genetic relationship to spindle cell lipomas and angiofibromas continuing to evolve. Their surgical management has not progressed, and local excision remains standard. Observation is poorly studied despite the lesion’s benignity and very low recurrence rates.  

### 2.3. Fibromatosis/Desmoid Tumor

Fibromatosis or desmoid fibromatosis was first mentioned in 1832 by Macfarlane, but the term desmoid tumor was not used until 1838 [26]. Desmoid tumors are defined by the World Health Organization as “clonal fibroblastic proliferations that arises in the deep soft tissues and is characterized by infiltrative growth and a tendency toward local recurrence but an inability to metastasize [27]”. In the breast, it is thought to arise from the pectoralis fascia or Cooper’s ligaments [28].

Desmoid tumors account for 0.2% of primary breast tumors [28]. They occur more frequently in pre-menopausal women [28,29]. Some cases, termed “cicatricial fibromatosis” are the result of trauma [28] or breast implants [29] through myofibroblastic stimulation [28]. The association between hormone stimulation and steroids is controversial [29]. Most are sporadic and associated with CTNNB1 mutations, a gene that encodes beta-catenin. Approximately 8–10% of breast fibromatosis is a manifestation of Familial Adenomatous Polyposis (FAP), a syndromic mutation in Adenomatosis Polyposis Coli (APC gene). Young patients (<40 years) with fibromatosis should be screened for FAP. The APC mutation and CTNNB1 mutations are mutually exclusive [28]. Recently, different mutations in CTNNB1 have been noted for different locations and may also play a role in biological behavior [30]. 

They present in the fourth or fifth decade of life [31] as firm, painless, and often mobile masses [29]. Skin changes and nipple retraction are common [28,29] whereas detection by screening imaging is less common [31]. They are multiple in 10% (and FAP should be excluded in these patients) [30]. 

Sonographically, they appear as poorly defined hypoechoic masses with posterior shadowing and echogenic rims. On mammography, they mimic breast cancer as high-density spiculated lesions [29]. Calcifications are rare [29] and the lesions can be occult [31] perhaps related to the young age of most patients. MRI findings are heterogenous with moderate to strong enhancement. One report suggested that MRI findings tend to report larger sizes of masses as compared to US and MG [31]. Desmoid Tumor Research Foundation (DTRF) guidelines recommend MRI as the primary diagnostic and surveillance modality for fibromatosis [27]. PET scans have shown promising results in evaluating responses to medical management and selecting those who should continue the selected regimen [32]. 

Core needle biopsy (CNB) is generally sufficient for diagnosis [30]. In Boland’s review of 16 patients, 3 CNBs were inconclusive. The DTRF update on fibromatosis does not recommend excisional biopsy upfront for confirmation, but CNB specimens should be reviewed by expert soft tissue pathologists given the high rate (30–40%) of misdiagnoses [30] and excisional biopsy performed only if it remains inconclusive [27].

**Figure 6:**
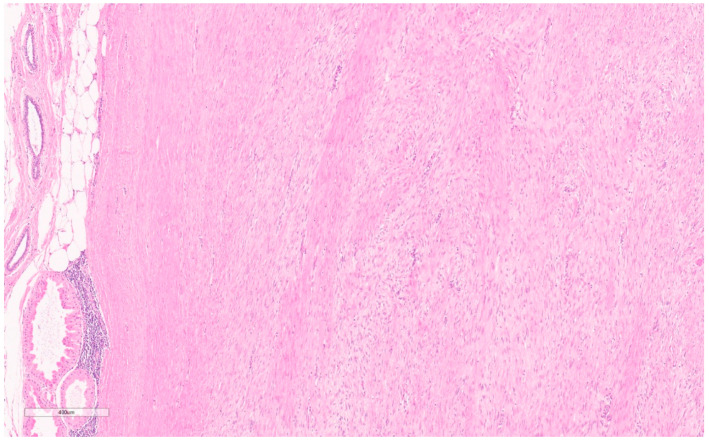
Fibromatosis, hpf, peripheral lymphoid aggregates, and fat entrapment.

Macroscopically, fibromatosis is rubbery, nodular, gray-white, and poorly vascularized [29,30]. Microscopically, fibromatosis demonstrated long spindle cells arranged in interlacing fibroblastic bundles and variable fibrosis. They have irregular “tentacle-like” margins infiltrating the breast parenchyma (see Figure 6) [28,30]. Cells are bland with hyperchromatic nuclei and frequent nucleoli. Mitotic figures are rare. Lymphocytic aggregates are often present at the periphery. Nuclear atypia and mitoses should raise suspicion of fibromatosis-like metaplastic carcinoma [28]. 

**Figure 7:**
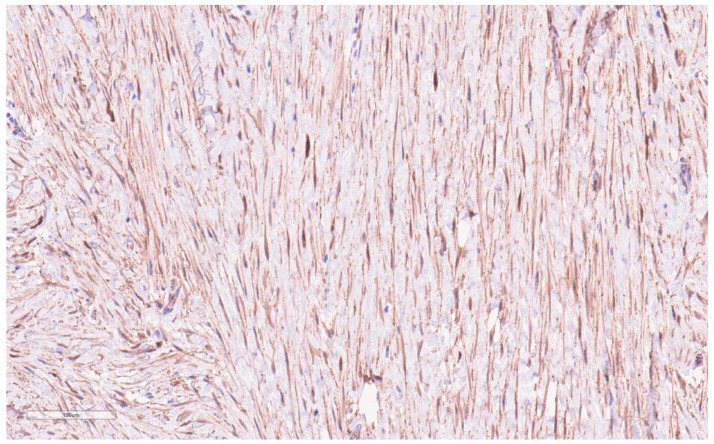
Fibromatosis, IHC for beta-catenin showing nuclear and cytoplasmic staining.

By IHC, cytokeratins, p63, ER, and PR are generally negative, whereas nuclear beta-catenin (see Figure 7) and actin are often positive. Variably positive markers include desmin, S100, and CD34 [28]. Beta-catenin mutations that lead to accumulation of the beta-catenin protein are present in 80–90% [28,30], which can confirm this diagnosis, though notably it may also be seen in Phyllodes tumors, metaplastic carcinoma, and other spindle cell lesions [28]. 

There has been increasing interest in genetic mutations as predictors for recurrence. For example, there is a correlation noted between beta-catenin S45F mutations and increased local recurrences. This subtype also showed the highest progression arrest rate when given imatinib (85%) compared to other wild-type mutations. These mutational analyses may help predict recurrence and response [30]. 

Surgery was standard of care until at least 2000 [30]. Papers as recent as 2013 recommend surgical excision despite local recurrence rates of 24–77% [29]. The infiltrative growth pattern leads to larger margin sizes and larger resections. With high recurrence rates and re-excisions, surgical management can lead to significant disfigurement [29]. The lack of margin status impact on recurrence rates prompted a re-evaluation of surgical management [30,31]. 

DFTR 2018 consensus guidelines do not recommend surgery as the standard of care [27], with a shift in the paradigm towards active surveillance and medical management. All cases should be discussed by a soft tissue tumor multidisciplinary tumor board (MDT) [27,28]. 

With active surveillance for extra-abdominal desmoids, one retrospective series (*n* = 216) reported that, with observation, 20% regressed, 5% converted to the need for surgery and 51% converted to the need for alternative medical strategies [33]. If possible, the lesion should be surveilled for 1–2 years with evidence of consistent progression prior to proceeding with alternate management. Surveillance would ideally be conducted every 3–6 months with an MRI or a CT if MRI is not possible [27]. There is currently no evidence to support the use of Positron Emission Tomography (PET) scans in the surveillance of DTs [27]. In the case of progression, management is site-specific [27]. In breast and chest wall cases, the DFTR recommends beginning with medical therapy [27]. Medical therapy can involve (1) non-steroidal anti-inflammatories (NSAIDs), (2) hormone therapy, (3) chemotherapy, usually methotrexate (MTX)-based, or (4) tyrosine kinase inhibitors (TKIs) [27]. 

The benefit of hormone therapy (tamoxifen or tomtirene) with or without Non-Steroidal Anti-Inflammatories (NSAIDs) is controversial. Supporting evidence comes primarily from small retrospective trials that have shown response rates up to 25% [34]. A prospective phase 2 trial using tamoxifen and sulindac in adolescents with desmoids at all locations did not demonstrate any benefit and demonstrated a 30% incidence of ovarian cysts in pre-menopausal women [35]. The DFTR consensus found insufficient evidence to support either treatment [27].

Tyrosine kinase inhibitors, including imatinib, sunitinib, sorafenib, or pazopanib, have recently revolutionized the management of desmoid tumors and are often considered the first line in American centers. Notably, compliance can become an issue, as the duration of therapy is generally approximately one year long. There is prospective evidence supporting the use of imatinib with reported stabilization of 60–80% but lower regression rates (5–15%). Sorafenib has also shown promise through retrospective review, with higher stabilization (70%) and regression (25%) rates. An American Randomized Control Trial (RCT) (*n* = 87) has demonstrated increased progression-free survival with sorafenib. [36] Patients should be included in clinical trials where possible [30]. Finally, a recent RCT (*n* = 142) from France that was presented at the European Society for Medical Oncology (ESMO) Conference in 2022 demonstrated the benefit of nirogacestat with a demonstrated 71% lower risk of disease progression on average [37].

Chemotherapy usually involves low-dose methotrexate with vinorelbine [38]. This is the first line of medical management at our center. Tumor response continues after the end of therapy and overall response rates for all anatomic locations are in the realm of 35–40%, with long-term control in 50–70% of those who respond [27,38]. Chemotherapy can be repeated in responders if the tumor recurs.

As a last resort, an alternative chemotherapy regimen is anthracycline-based, similar to sarcoma regiments with reported response rates of 37% but only evaluated in two retrospective series [27,39,40]. This regimen carries higher toxicities than an anthracycline—pegylated liposomal doxorubicin may decrease toxicity compared to traditional anthracyclines. Similarly, however, there are only two retrospective series evaluating this with response rates of 35% [27,41]. 

If medical therapy fails, the next step in the DFTR algorithm for truncal fibromatosis (including breast) is to (1) change the medical therapy, (2) consider radiation, or (3) consider surgical management. 

Radiation has high reported local control rates. Delivery is challenged by the large sizes and risk to normal tissue of surrounding structures as well as the risk of secondary neoplasms [42]. Radiation can be considered after medical therapies have been exhausted and though it can be considered primarily, ideally it would be for sites where local control is difficult to achieve surgically, for patients who are not surgical candidates, or for recurrences. The presence of S45 mutations may increase recurrence rates, but this risk may be offset by the administration of radiation [30]. Remission rates for radiotherapy alone are 15% for all desmoid fibromatosis (including extra-mammary sites) [31]. 

Surgical excision of fibromatosis is not advisable but remains an option if procedural morbidity would be low and the lesion is progressive on >2 scans or particularly symptomatic, with the caveat of frequent locoregional recurrences and with caution to repeated excisions causing significant cosmetic deformities [28]. Margin status has not been shown to influence recurrence, whereas beta-catenin mutation presence and bodily location does (with the breast being at lower risk of recurrence) [31]. In patients with complete macroscopic resection, undergoing primary resection, disease-free survival was only 76% in one series (*n* = 203) [43].

#### Summary of Updates

The management of mammary desmoid fibromatosis has changed dramatically over the last twenty years. Surgical management is now discouraged. Medical management, including low-dose methotrexate and vinblastine, is preferred and TKIs are gaining popularity and have shown success in small RCTs.

### 2.4. Inflammatory Myofibroblastic Tumors 

Inflammatory Myofibroblastic Tumors (IMTs) were first described in the lungs by Bunn in 1939 and the first breast case was described in 1988 by Pettinato [44]. They have previously been considered a subgroup of inflammatory pseudo-tumors (IPTs) but despite interchangeable use in the literature and similar morphology, they have different clinicopathologic features [45]. They were officially defined by the WHO in 2022 as myofibroblastic, spindle cell proliferations with background inflammatory changes and intermediate malignant potential [28,46]. 

Mammary IMTs present as painless, slow-growing nodules [28] in young women with two case reports in men [45]. They have a wide anatomic distribution, though breast case reports are extremely rare. Pathogenesis is unknown; they were thought to represent inflammatory reactions, but the recently discovered gene mutation in the anaplastic lymphoma receptor tyrosine kinase gene (ALK) suggests a more neoplastic origin [47]. 

Ultrasound and mammography are non-specific and descriptions are limited to case reports [48,49] though generally lobulated masses that are hypoechoic on the former and hyperdense on the latter. Calcifications are not uncommon [48,49]. Two reports with MRI descriptions suggest a typical appearance to be a single mass, with unclear, lobulated boundaries, rapid enhancement, and washout [49,50]. 

**Figure 8:**
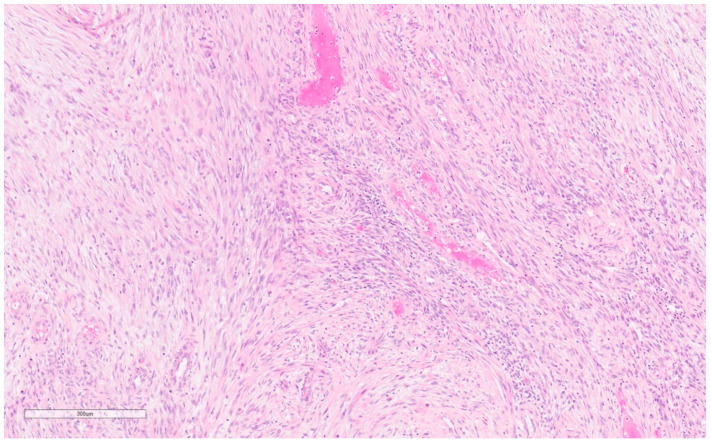
Inflammatory Myofibroblastic Tumor, myofibroblasts, and inflammatory cells.

Macroscopically, they are fleshy tumors with areas of calcification, necrosis, and hemorrhage [28]. On CNB, they have variable proportions of spindle cells and stromal cells with inflammatory infiltrates (lymphocytes, plasma cells, eosinophils) (see Figure 8) [46]. Plump myofibroblasts form short fascicles in a myxoid or hyalinized background with inflammatory infiltrate [46]. Epithelioid variants have prominent neutrophilic components and abundant myxoid stroma. They may demonstrate mitoses, but have no atypia or pleomorphism—differentiating them from sarcomas [28]. 

On IHC, IMTs stain positive for smooth muscle actin (SMA), mammary serum antigen (MSA), calponin, desmin, and occasionally for cytokeratins. They are negative for p63, CD34, and S100 [28]. If the diagnosis is suspected, ALK1 and ROS1/NTRK3 stains should be performed. Diffuse cytoplasmic ALK1 will be expressed by 50–60% [47] with epithelioid variant showing nuclear membrane or perinuclear staining. For ALK1-negative cases, molecular studies for ROS1 and NTRK3 are often helpful [47]. Diagnosis often requires evaluation by an expert pathologist [28]. 

The rarity of this lesion perpetuates a poor understanding of their natural history. Given there are reports of metastases (though rare) [45,47,49] and malignant behavior, wide local excision to negative margins is recommended [45]. Axillary management is undefined [45] though no axillary involvement has ever been reported and the more sarcomatous nature may mean they are unlikely to spread lymphatically. Mammary recurrence rates are reported as high as 25% [49]. Chemotherapy, radiotherapy, and immunotherapy have few reports, especially in the breast. One case report in metastatic, extra-mammary IMT demonstrated complete radiographic tumor response at 1 year with ALK inhibitors crizotinib and alectinib [51]. These agents should be considered if an ALK mutation is identified [46]. Though understudied, this is recommended if aggressive breast disease is encountered. 

#### Summary of Updates

Though only recently described, understanding of IMTs has been slow to progress and has been aided primarily by the amalgamation of international case reports. IMTs remain non-specific on imaging and local excision remains the standard of care. Newly detected genetic mutations in ALK1 have helped make pathologic diagnosis more straightforward with biopsy and provide a target for immunotherapy in aggressive diseases, though the latter remains understudied.

### 2.5. Dermatofibrosarcoma Protuberans

Dermatofibrosarcoma protuberans (DFSP) was first described in 1924 by Darier and Ferrand and acquired its official name a year later in 1925 by Hoffman [52]. DFSP is a rare neoplasm originating from dermal fibroblasts. DFSP has a predilection for the upper extremities of patients in their third and fourth decades of life [53]. A history of trauma has been proposed as a risk factor but has only been noted in 10–20% of cases [54,55]. 

DFSP presents as an indolent, painless skin-colored plaque or papule centered in the dermis [28,52]. Sonographically, lesions are oval, parallel to the skin, and heterogeneous. Mammographically, they are hyperdense with no calcifications [28]. Pre-operative imaging is not sensitive or specific at estimating the depth of invasion in subcutaneous tissue. 

**Figure 9:**
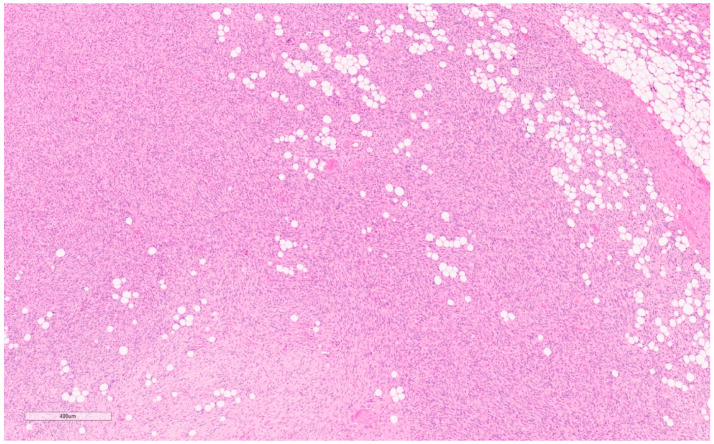
Dermatofibrosarcoma protuberans, honeycombing of fat.

Macroscopically, they are unencapsulated, well-circumscribed masses of white fibrous tissue with some degree of gelatinous or mucinous appearance [28]. Though mammographically they may appear to be within the breast parenchyma, microscopically they will be contiguous with skin and are generally centered in the dermis or subcutis [28]. They appear as hypercellular fascicles of spindle cells with a storiform architecture infiltrating the subcutis with a “honeycomb” pattern (see Figure 9) [28]. They have a classic appearance on frozen sections consisting of (1) cigar-shaped nuclei (2) cartwheel pattern of nuclei arrangement, and (3) fibrotic stroma [56]. 

**Figure 10:**
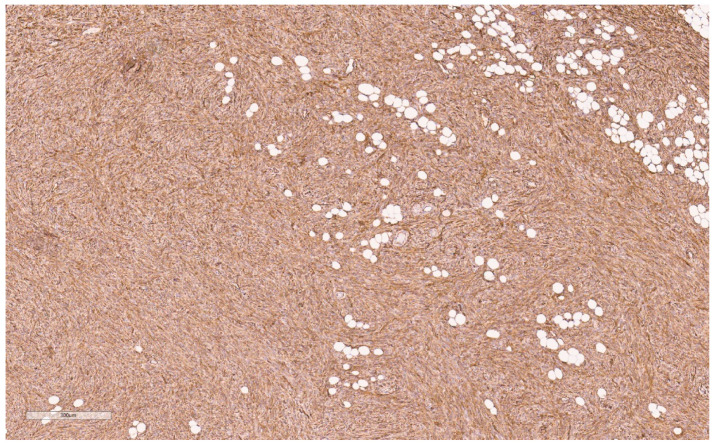
Dermatofibrosarcoma protuberans, IHC staining positive for CD34.

On IHC, tumors stain positive for CD34 (see Figure 10) and WT1 and stain negative for keratin, S100, and muscle markers (except in myoid differentiation). Lack of CD34 staining with nuclear atypia or increased mitotic activity should raise suspicion of malignancy [28]. More than 90% contain a classic translocation (17:22) from a fusion of COL1A1 with PDFGB. This mutation leads to the perpetual activation of PDGFB [57]. A variant mutation of COL6A3-PDFGB has been shown to have a predilection for the breast [57]. 

The standard of care is wide local excision [52] and tends to recur without aggressive local management [28]. Exact margin recommendations are undefined, but microscopically, tumors extend well beyond an appreciable clinical extent [52]. There is controversy around the use of Moh’s micrographic surgery for DFSP and our group does not consider it useful in mammary DFSP [58]. One systematic review, focusing on extra-mammary DFSP demonstrated lower local recurrence rates [53,55,59] but it has not been reported in the breast, it is expensive, creates more complex wounds for closure, and has longer operative times. Additionally, mammary fat content makes frozen sections more technically challenging. 

They have limited sensitivity to chemotherapy and radiation (which has no utility outside of malignant transformation). Imatinib can be considered in the neoadjuvant context for borderline resectable or recurrent cases [58]. Mutation profiles have demonstrated some ability to predict encouraging results for tumor shrinkage with imatinib given in this setting [52,60]. Radiation can be considered in high-risk cases (high mitotic index or positive margins where the margin cannot be re-excised) [52]. 

Metastases occur only in cases with fibrosarcomatous (FS) progression (FS-DFSP). Approximately 10–14% contain FS progression and 1–4% metastasize (which usually occurs after local recurrence) (Dimas). Metastases are hematogenous only, negating the need for axillary staging. Recurrence rates are reported at 2–25% [53,58]. In addition to margins, recurrence rates are related to the depth of invasion, anatomical location, and FS status. Axillary staging is therefore not indicated [52,58,61]. Recurrences have been reported more than 5 years from diagnosis and long-term follow-up is indicated, though the exact duration is also undefined [52]. 

#### Summary of Progress

New genetic markers have been identified in the last 20 years, including COL6A3-PDFGB which has a predilection for the breast. The minor successes in the retrospective series seen with Moh’s surgery at extra-mammary sites are not applicable to breast cases. On the contrary, recent successes with imatinib in extra-mammary DFSP may be extrapolated to the breast, though is still currently understudied. 

## 3. Benign Smooth Muscle

### 3.1. Leiomyoma

Leiomyomas are benign tumors of the smooth muscle that are most common in the small bowel and uterus [62]. Mammary lesions are rare, accounting for <1% of breast tumors [63]. They can be classified by location as follows:

(1) Cutaneous: primarily a dermatological diagnosis [62], outside the scope of this review.
(2) Nipple Areolar Complex (NAC): Nipple leiomyomas were first described in the 1850s by Virchow [64,65]. They are related to the presence of smooth muscle cells in the nipple (muscularis mamillae) [65].
(3) Intra-parenchymal: Least common of the three lesions first described in 1913 by Strong et al. [64,66]. Histogenesis in this location is unclear; there are theories that they arise from smooth muscle cells within blood vessels [67].

Since their description in 2012, studies have identified 21 cases of nipple leiomyomas [64]. Similarly, from description until 2018, 30 cases of intra-parenchymal leiomyomas have been identified [68]. They are most often diagnosed in the fifth to sixth decade of life as small, slow-growing masses that are frequently painful, especially with cold and palpation. Painful episodes are thought to be related to smooth muscle contraction. They are less commonly screen-detected [67].

Mammographically, they are homogenous dense lesions with well-defined margins and no calcifications [64,69]. They can appear as an enlarged nipple [64] or as spiculations extending from the nipple into the subareolar tissue [64,65]. Sonographically, they appear solid, homogenous, hypoechoic, and well-circumscribed with variable posterior shadowing [64,70]. On MRI, they are well-circumscribed, whorl-appearing lesions with low T1 signal intensity, low to intermediate T2 signal intensity, gradual homogenous enhancement, and peripheral rim enhancement [64,67]. Tumor degeneration can provide heterogeneity [67]. 

Macroscopially, leiomyomas are fleshy pale white lesions [67]. Histologically, they appear as spindle cells in “cigar-shaped” blunt-ending nuclei with eosinophilic cytoplasm [28]. 

On IHC, leiomyomas are positive for desmin, smooth muscle actin, and h-caldesmon and negative for CD34, p63, S-100, and keratin [28,67]. If ever multiple leiomyomas are present, patients should be tested for hereditary leiomyomatosis renal cell carcinoma HLRCC syndrome (fumarate hydratase germline mutation) [71]. Suspect a leiomyosarcoma with cytologic atypia, >2 mitoses/10 high-power fields, necrosis, atypical mitoses, infiltrative growth pattern, and vascular invasion [67]. In 2022, an intermediate subtype of atypical leiomyomas was described. Definitions for atypical leiomyomas exist for uterine leiomyomas; however, the rarity in the breast makes defining this entity challenging [72].

The natural history of leiomyomas in the breast is not well studied. Similar to extra-mammary locations, they are considered indolent and non-aggressive [73]. The majority of described cases have been excised, aiming for negative margins, with no recorded recurrences or malignant transformations [63,68,70]. Some believe enucleation is adequate [73]. It has been proposed that typical lesions in the NAC could be observed unless they are symptomatic, cause cosmetic problems [28], or have diagnostic dilemmas.

#### Summary of Updates

International case reports of these rare lesions have improved classic descriptions for easier radiologic and pathologic identification. However, the ongoing poor understanding of natural history has not allowed practitioners to stray from surgical excision as the mainstay of management.

## 4. Peripheral Nerve Sheath Tumors

### 4.1. Neurofibroma

Neurofibromas were first described in 1882 by von Recklinghausen and first noted in the breast in 1981 by John Sherman [74,75]. They are defined as benign peripheral nerve sheath tumors arising from the endoneurium, or the connective tissue of nerve sheaths [76].

Lesions can be associated with neurofibromatosis type 1 (NF1, aka von Recklinghausen disease), an autosomal dominant genetic disorder. Most lesions (90%) are sporadic but also tend to have intra-lesions NF1 mutations [77,78]. Most neurofibromas affect the head, neck, and extremities, with only a few case reports of mammary neurofibromas in the absence of NF1 [75,79]. In NF1, neurofibromas within the breast account for only 3.5% of all tumors [80].

Sporadic neurofibromas tend to occur in the third or fourth decade of life without gender predilection [28,78] as solitary, slow-growing, and painless lesions in superficial tissues. In the breast, there is a predilection for the NAC or the pectoralis fascia [78]. There are three described subtypes; two can be found in the breast, as listed below. The third subtype, plexiform lesions, occur along deep nerve roots, are pathognomonic for NF1, and are outside the scope of this review with only one case report of occurrence in the breast (involving the breast and axilla with a possible origin from the brachial plexus) [80].

1. Localized: Localized neurofibromas account for 90% of these lesions [78,79,81]. Clinically, they are lobular skin-colored lesions with pathognomonic “button-hole sign [81]”.
2. Diffuse: these are locally invasive, growing as plaque-like lesions in the subcutaneous tissue [77].

Sonographically, localized lesions appear as superficial, circumscribed nodules that are hypoechoic and resemble cysts [79] or fibroadenomas [78]. Diffuse lesions can appear hyperechoic, containing multiple interconnected hypoechoic tubular structures and lesion extent is often difficult to determine [81]. Mammographically, they are hyperdense, well-circumscribed lesions that can be missed given their propensity to occur posteriorly (retro-glandular fat or pectoralis fascia) [78]. On MRI, neurofibromas are hypo- or iso-intense on T1 and hyperintense and heterogenous on T2 with lack of or gradual enhancement [75]. Heterogeneity is related to the composition of the mass matrix from myxoid and fibrous components [78]. Signs of malignant transformation include rapid enhancement with washout, larger size, peripheral enhancement, intra-tumoral lobulation, and peritumoral edema. Atypical neurofibromas may be more PET-avid [82].

**Figure 11:**
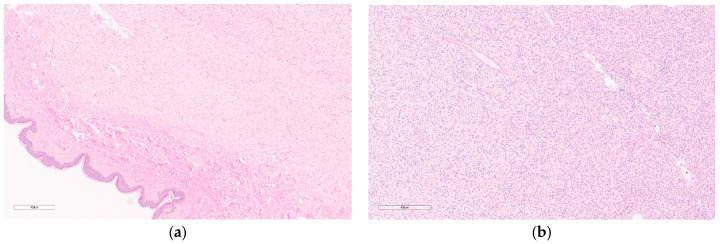
Neurofibroma, (**a**) with overlying skin; (**b**) on excision with comma-shaped nuclei and “shredded-carrot-like” stroma.

Macroscopically, they are unencapsulated, well-circumscribed gray firm masses with gelatinous cut surfaces and transected nerve fibers attached [77]. Microscopically, they appear as bipolar spindle cells with bland, comma-shaped, or wavy serpentine nuclei with smudgy chromatin and “shredded-carrot-like” collagenous stroma (see Figure 11) [78].

They stain positive for S100 and SOX10 and variably stain positive for CD34 [78]. Malignancy should be suspected of a loss of H3K27me3 or increased cellularity, fascicular growth, nuclear pleomorphism, mitotic activity, and necrosis [78]. In 2017, Miettenen et al. published a consensus report with pathologic criteria for challenging neurofibromas to eliminate the ambiguous term “atypical neurofibroma” and propose a new entity of Atypical Neurofibromatous Neoplasms of Uncertain Biological Potential (ANNUBP) [83]. The overall goal of these criteria is to promote an understanding of which lesions require multidisciplinary management [83,84].

The current literature on these rare tumors has primarily involved surgical excision, and this is generally accepted as the mainstay of management [77] despite the low risk of malignant transformation [85]. Their natural history is not understood well enough for observation to be recommended. At a minimum, symptomatic lesions or lesions with any suspicion of atypia or malignancy should undergo surgical excision [78].

The prognosis is very good with few loral recurrences, especially in patients without NF1. Margins have been shown to impact recurrence rates, especially in atypical lesions [86]. The rarity of this location for neurofibromas has prompted the recommendation for all mammary neurofibroma patients to be referred for genetic counseling [78]. NF1 patients have an 8–13% lifetime risk of malignant peripheral nerve sheath tumors and, interestingly, also have twice the lifetime risk of breast cancer [78]. This is especially notable in young women in whom the risk of breast cancer in NF1 patients in 8.4% under 50 years old. NCCN recommends NF1 patients begin annual screening tomosynthesis (with consideration of MRI as an alternative between 30 and 50 years old) beginning at 30 years old [87].

#### Summary of Updates

New terminology (ANNUBP) and pathological criteria have recently been defined for borderline lesions. Screening recommendations have been recently updated and include genetic counseling for all mammary neurofibromas and intensive breast cancer surveillance for NF1 patients.

### 4.2. Granular Cell Tumors

Weber and Virchow first described granular cells tumors (GCTs) of the tongue in 1854 and Russian pathologist Abrikossoff then described them as “myoblastomas” in 1926, hence their pseudonym, Abrikosoff tumors [88]. With the advent of immunohistochemistry (IHC), GCTs are now thought to arise from Schwann cells with neuroectodermal differentiation [89].

GCTs involve the breast in 5–8% of cases [90] and malignancy occurs in 2% [91]. Multiple GCTs occur in 5–10% of patients and are associated with Noonan syndrome, neurofibromatosis type 1, and LEOPARD syndrome suggesting a possible relation to abnormal RAS/MAPK pathway (mutation seen in all three syndromes) [92]. GCTs present as painless, firm masses, commonly in the upper inner quadrants (related to the distribution of the cutaneous sensory cutaneous nerve) [91]. A quarter of women present through screening [91].

Mammography demonstrates spiculations and architectural distortions [93] usually without calcifications. Sacranelo et al. reported no calcifications in any GCTs reviewed, suggesting that their presence may contradict the diagnosis [94]. Sonographically, they are hypoechogenic with irregular borders. One author has postulated that adding elastography and contrast-enhanced ultrasound (US) to these routine investigations may help differentiate these lesions from breast cancer, but these have not been studied in this context [95]. On MRI, granular cell tumors have intermediate-low homogenous T1-weighted signal intensity and are difficult to visualize on T2 images [91,94,96]. They are variably enhanced with some reporting rim enhancement [91,94,96]. In extra-mammary locations, positron emission tomography (PET) can differentiate GCTs from malignant tumors as they will not show increased metabolic activity and have standardized uptake values (SUVs) of 1.8 (SUV malignant GCT = 2.8) [91].

**Figure 12:**
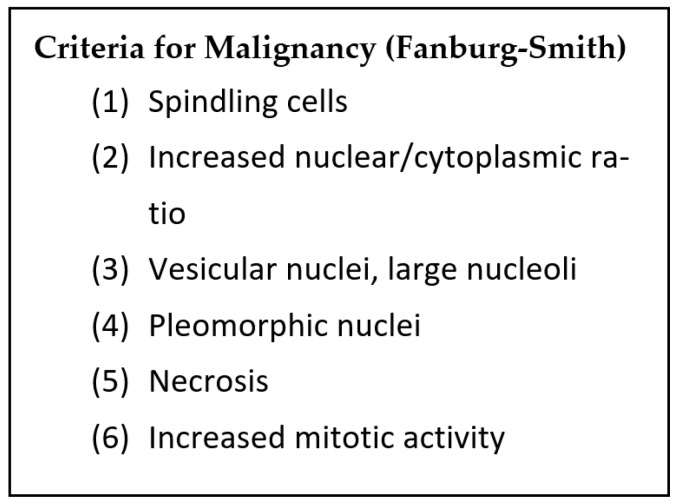
Fanburg-Smith Criteria for Differentiating Malignant and Benign Granular Cell Tumors.

Macroscopically, they are white and spiculated lesions [28]. Microscopically, they are composed of infiltrating sheets of polygonal bland cells with well-defined cell borders and abundant eosinophilic granular cytoplasm [90]. They stain with Periodic Acid Schiff (PAS) (diastase resistant) and IHC positive for S-100, SOX10, and CD68 [92]. If ≥3 of the criteria defined by Fanburg-Smith (see Figure 12) are present, then the lesion is considered malignant and if 1–2 of the criteria are present, it is considered atypical [92].

In 2018, Pareja et al. performed whole exome sequencing of benign and malignant GCTs and identified inactivating mutations of ATP6AP1 and ATP6AP2 in 49–74% [92,97,98,99]. In vitro inactivation impairs the V-ATPase gene complex in Schwann cells and leads to the formation of GCTs [97]. Dehner et al. demonstrated in 2022 that multifocal benign GCTs in the same patient are molecularly distinct, whereas multifocal malignant GCTs, which are much less common, harbor identical mutations [99].

Surgical-wide local excision with negative margins (no tumor on ink) [91] is the ideal treatment, though it carries a risk of local recurrence. [91,100]. This risk is 2–8% with negative margins and 20% with positive margins [101]. One case of recurrent benign GCT has been described where the lesion was observed and stable for two years [84]. Natural history is not understood well enough to recommend this, nor to recommend surveillance guidelines. Late recurrences in case reports have been noted [91] at 2 years and 4 years. Some have recommended surveillance until 10 years [91] though, in mammary-specific cases, the latest identified recurrence has been at 4 years [101].

#### Summary of Updates

In the last decade, the genetic profile of GCTs has been delineated, though the clinical significance of the identified mutations remains unclear. Similarly, case reports may support GCT observation, but natural history requires definition before management or surveillance recommendations can be made.

### 4.3. Schwannoma

Schwannomas were first described in 1910 by Verocay and first described in the breast in 1973 by Collins and Gau [101,102]. They are benign tumors arising from differentiated Schwann cells, i.e., cells that form the myelin of peripheral nerves, facilitating impulse transmission [103].

Most peripheral schwannomas affect the head, neck, and extremities; mammary schwannomas account for only 2.6% of schwannomas [103]. A 2011 report identified only 27 cases in worldwide literature [103] and in our literature search, we identified a further 17 cases in women [104,105,106,107,108,109,110,111,112,113,114,115,116,117] and 2 male cases [118,119]. Sporadic cases account for 90% of cases [84], whereas the rest are syndromic and associated with neurofibromatosis type 2 or familial schwannomatosis [84]. They present as solitary lesions along the course of a nerve [84]. Pain to palpation occurs in 95% of patients but in only 5% of patients at rest [104]. There have been five reported cases of axillary schwannomas in breast cancer patients which may mimic metastatic disease [120,121].

Breast imaging for schwannomas is not well studied. At extra-mammary sites, they are reported as being variable sonographically, given their varying composition and areas of degeneration and hemorrhage [122]. Mammographically, they can be occult or present as well- or ill-defined masses [122]. MRI findings are often isointense on T1 and heterogenous on T2 with a hypointense rim [123,124]. There is a case report of PET positivity consistent with the common FDG uptake seen in extra-mammary schwannomas [107]. Intense radiating pain during a biopsy is a diagnostic clue. FNA results are variable depending on whether the needle goes through an Antoni A or B area (see below). CNBs are generally required to identify Verocay bodies [115]. Excisional biopsy is often needed [120].

Macroscopically, they are well-circumscribed, encapsulated, white/tan, and multilobulated [114]. Similar to radiographically, they are microscopically diverse and related to cystic, hemorrhagic, and degenerative areas. Mitoses and atypia may occur, but necrosis is always absent. Schwannomas have five classic features: (1) varying degrees of encapsulation, (2) lymphoid cuffs, (3) clusters of hyalinized vessels, (4) alternating compact hypercellular (Antoni A) and myxoid hypocellular (Antoni B) regions, and (5) nuclear palisading (Verocay bodies) [119]. All schwannomas will stain positive for S100 and SOX10 [84]. Malignant peripheral nerve sheath tumors should be suspected with necrosis, loss of p16 expression, fascicular growth, and perivascular bulging [84]. Whether syndromic or not, many have NF2-inactivating mutations.

Surgical excision is the mainstay of management [104] and is considered curative [120], though research is sparse. Enucleation is likely sufficient, simple, and minimally co-morbid (though can cause paresthesias). At extra-mammary sites, this landscape is changing. There have been promising results for watch and wait approaches [125] and gamma knife surgery [126]. Vacuum-assisted excision has been proposed to minimize intervention for breast schwannomas but has not been studied [114]. These all may be upcoming options, though less relevant in breast tumors given the low morbidity of lumpectomies. Schwannomas can locally recur but have no ability to metastasize or invade locally. Malignant transformation is rare [84], with four cases reported in English literature prior to 2005 [122].

#### Summary of Updates

Case reports of mammary schwannomas have increased, but most evidence remains extrapolated from extra-mammary sites. Similarly, minimally invasive treatments that are unstudied in the breast have had some success at extra-mammary sites and may be forthcoming for breast patients.

## 5. Benign Adipocytic

### 5.1. Lipoma

Lipomas are defined as benign tumors originating from adipocytes. They are the most common mesenchymal tumor of the body, 20% of which occur on the chest wall [127,128].

The pathogenesis of lipomas is unclear though has been hypothesized to be related to trauma-induced cytokine release triggering pre-adipocyte cell differentiation [127]. Multiple lipomas are present in 5–10% of people, some of which are syndromic. Syndromes include Proteus disease (AKT1 oncogene mutation), Dercrum disease, familial multiple lipomatosis and Madelung disease, Gardner syndrome, Multiple Endocrine Neoplasia type 1, and Cowden syndrome [127]. A mutation in HMGA2-LPP has been described in solitary lipomas [127].

They present as soft slow-growing painless masses usually noted in the fourth to sixth decade of life. Risk factors include obesity, dyslipidemia, and diabetes (Kolb). Protease inhibitors used in HIV can induce lipomas [127]. Imaging appearances can be sufficiently specific for diagnosis.

In extra-mammary locations, imaging is not always required if the lesion is classic on exams. In the breast, imaging is indicated. Mammographically, they are radiolucent with a thin radio-opaque capsule [128]. They can have calcifications related to fat necrosis. Sonographically, they are hypoechoic and avascular [127].

**Figure 13:**
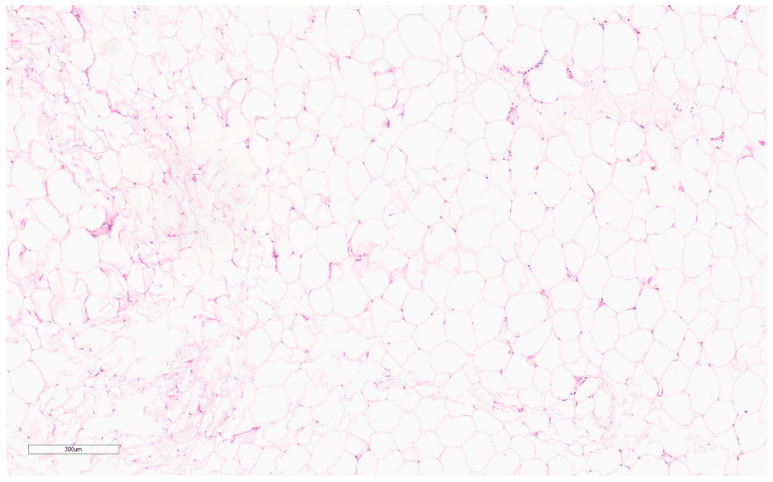
Lipoma, normal adipocytes with small nuclei and interspersed fibrous septae.

If classic radiologic appearance is lacking, then tissue diagnosis should be pursued [129]. CNB is preferred as a diagnosis can be challenging due to the presence of substantial adjacent adipocytic tissue [130]. Microscopically, lipomas appear as normal adipocytes with small nuclei (see Figure 13). They also contain interspersed fibrous septa with blood vessels [127]. They have many histologic subtypes that are often difficult to differentiate from liposarcomas including spindle cell lipomas, myelolipomas, angiomyolipomas, fibrolipomas, pleomorphic lipomas, ossifying lipomas, hibernomas, and chondroid lipomas. Liposarcomas have coarser vacuoles and scalloped hyperchromatic nuclei [127].

Lipomas can frequently be observed. Conservative measures such as weight loss may help may lipomas become less conspicuous. Alternatively, two or three injections of deoxycholate have shown a decrease in 75% of lesions [131]. Removal of a lipoma to rule out a liposarcoma can be considered depending on size (>10 cm, rapid growth, pain, fixation to underlying structures) [127]. Outside these indications, cosmesis is the most common reason for surgical excision. Conventionally, the entire capsule should be removed during excision. There is research ongoing into more minimally invasive techniques for removal, including liposuction [132]. A series of 30 patients using minimally invasive techniques (a combination of liposuction, minimal incisions, tunneling, and “squeezing out” the lipoma) for multiple lipomas demonstrated excellent cosmetic results but recurrence in 5 patients [132]. Deoxycholic acid has been injected neoadjuvantly to limit the size of the resection and scar with some success [131]. Minimally invasive 1444 nm Nd:YAG (neodymium-doped yttrium aluminum garnet) laser was shown to be effective in a series of 60 patients [133]. This procedure is scarless and has minimal side effects; the most commonly observed being temporary edema and ecchymosis [133]. The prognosis is excellent with no metastases, but recurrences are possible when excision is incomplete [127]. 

#### Summary of Progress

Lipomas are benign tumors of adipose tissue that have been described for many years with minimal change to their management or diagnosis. Non-operative management is preferred if there are no concerns around diagnosis or cosmesis. In those being removed, interest is developing in minimizing surgical scars and morbidity through minimally invasive techniques described above such as liposuction with minimal incisions, deoxycholic acid injections, and laser therapies.

### 5.2. Angiolipoma

Angiolipomas were first described in 1960 by Howard and Helwig [134,135]. They are defined as tumors of mature adipocytes that also contain blood vessels. Pathologic criteria were proposed in 1974 for diagnosis, with lipocytes consisting of at least 50% of the lesion and evidence of angiomatous proliferation [136]. More recently, the angiomatous component has been found to compose 5–95% (median = 30%) of the lesions with more angiomatous lesions now termed cellular angiolipomas [134,137]. These account for 25% of angiolipomatous lesions [138].

Angiolipomas occur in the sixth decade of life [138,139], with cellular variants occurring later in the seventh decade [138]. They are more common on the trunk or forearm than the breast. They have been reported in men [134,140,141] and 55% of angiolipomas have been found to express androgen receptors [142], though they still have a female predilection. Angiolipomas are painful at extra-mammary sites [28,134,136] though breast lesions are often painless [138]. Their painless nature leads to a trend towards screen-detection, which may explain the later age of diagnosis as compared to extra-mammary sites [138]. Two thirds of extra-mammary angiolipomas are multiple in presentation, whereas breast lesions seem to be singular [138]. 

Radiographic characteristics vary by cellularity. In general, mammographically, they can be occult or present as well-circumscribed isodense masses [134,139]. Calcifications are generally absent, though have been reported [134,138]. Sonographically, they are well-circumscribed, homogenous, and iso to hyperechoic [134]. The largest series of angiolipomas to date by Kryvenko et al. reported that cellular angiolipomas were absent from all US but visible on mammography, whereas the inverse was noted in low cellularity angiolipomas [138]. One report of MRI findings demonstrated a low T1 signal intensity, high T2 signal intensity, and progressive contrast enhancement [143]. Purple skin discoloration, visibility on US, and larger sizes also raise suspicion for angiosarcomas [138].

**Figure 14:**
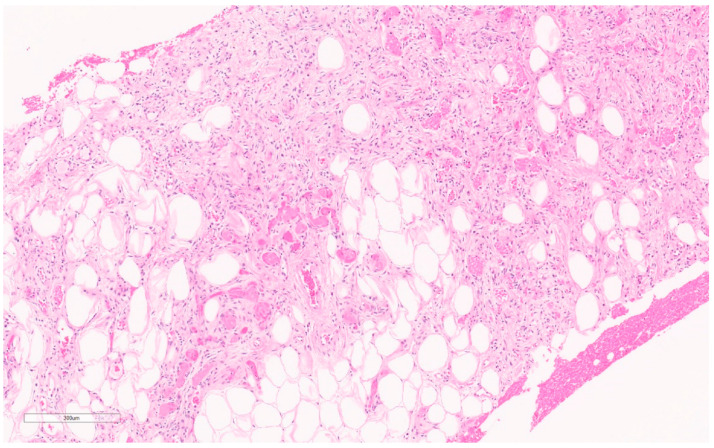
Angiolipoma, mature adipocytes with interspersed, uneven distribution of blood vessels in lobulated collections.

Macroscopically, this tumor is encapsulated, yellow with focal areas of gray and pink, usually <2 cm [134]. For tissue diagnosis, a core needle biopsy is usually sufficient [144]. Microscopically, there is a mature adipose component with branching capillary-sized vessels with hyaline microthrombi (see Figure 14) [28,138]. Lipomas will also contain small vascular branches, but the two are differentiated by distribution and quantity. In angiolipomas, the distribution of vessels is uneven with lobulated collections of capillaries at the lesion periphery, clustered with ≥ 3 capillaries in contact with one another, each lined with one layer of endothelium, and with no peripheral tufting [28,138]. Cellular angiolipomas may have mast cell infiltrates [138], spindle cell proliferation between capillaries, or mucinous changes in the interstitium [138]. Thrombosis is generally absent from other vascular breast tumors [138], including from angiosarcomas. If mitotic figures or blood extravasation are present, angiosarcoma should be suspected [138]. On IHC, they are positive for CD34, CD31, ERG, S100 and focally positive for smooth muscle actin.

With radio-pathologic concordance and adequate tissue sampling, excision is not necessary [28,138,144] despite being recommended as recently as 2016 [145]. These lesions can be observed with a currently undefined surveillance strategy. The cellular subtype of angiolipomas is too difficult to differentiate from low-grade angiosarcoma to leave in situ and excisional biopsy is indicated [138,146]. Again, there is no data for the surveillance of these lesions. There is no predilection for malignant transformation or recurrence, though follow-up data are sparse [138].

#### Summary of Updates

Cellular angiolipomas have been defined since their original description, with cellularity varying from 5 to 95%. Their management has moved towards conservative observation with limited data on surveillance and with excisional biopsy only if high-risk features are present.

## 6. Benign Vascular

### 6.1. Hemangioma

Mammary hemangiomas were first described by Image and Hake in 1847 and the first operative case was by Sutton in 1889 [147]. They are defined as benign vascular lesions consisting of a mass of blood vessels [148]. There have been many types of hemangiomas described, with the first classification system described by Rosen in 2001 [149]. The International Society for the Study of Vascular Anomalies (ISSVA) described a new classification system in 2018. This uses a structural differentiation of lesions and further differentiates between lesions with endothelial proliferation and lesions without proliferation. Within the proliferative group, lesions are classified as benign, borderline, or malignant [149]. The focus of this review is benign proliferative lesions of capillaries. Mammary hemangiomas can be perilobular, capillary, or cavernous, depending on the size of the involved vessels [150], with cavernous being more common in the breast [151]. Perilobular hemangiomas are always incidental and usually <2 mm in size. The subclassification between capillary and cavernous has minimal clinical significance [152].

They present in the sixth decade of life with a female predilection. As of 2018, there were 19 cases reported in men [144,148]. They are found in 11% of breasts post-mortem [153] and in 1% of mastectomy specimens [154]. Hemangiomas are typically small lesions < 2 cm that are detected incidentally on screening mammography [155]. They can appear as superficial palpable masses frequently with skin discoloration [144,153], though this should raise suspicion for angiosarcoma [154]. Size may also be a useful indicator of malignancy; benign lesions are typically <2 cm, whereas angiosarcomas are typically >2 cm [144]. Hemangiomas also tend to be more superficial than their glandular angiosarcoma counterparts [151].

On mammography, they have non-specific findings but are usually small micro-lobulated [153] and well-circumscribed without calcifications [150]. If calcifications are present, they are related to phleboliths or calcified thrombi [153]. Sonographically, they are variable with descriptors ranging from hypoechoic and well-circumscribed [150] to hyperechoic and ill-defined [153] to isoechoic with significant heterogeneity but smooth margins [148,155]. They are difficult to differentiate from complex cysts or fibroadenomas on US [150]. Some doppler US have shown high vascularity [150,153]. On MRI, they present as circumscribed masses with fibrous septa [153] with intermediate signal intensity on T1 and high signal intensity on T2 [150]. In contrast, the MRI will show fast enhancement with washout [150]. Overall morphology suggests benignity, but the washout patterns make it difficult to exclude angiosarcomatous lesions [150]. There have been recent studies on contrast-enhanced US (CEUS) differentiating benign and malignant solid breast lesions [156]. Some have suggested, supported by case reports, that these findings can be extrapolated [150] to vascular lesions, with homogenous, rapid early enhancement distinguishing hemangiomas from angiosarcomas (slow heterogenous and late enhancement).

**Figure 15:**
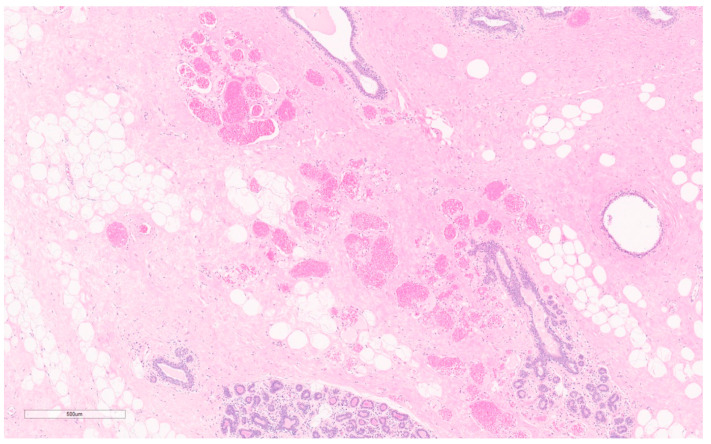
Hemangioma, area of dilated capillary-like vascular spaces with bland endothelium.

Macroscopically, they are well-defined, pigmented lesions. On biopsy, more excessive bleeding usually can be expected in these lesions [144]. FNA is often inconclusive, hypocellular, and considered inadequate to differentiate between benign and malignant vascular lesions [157]. On CNB, capillary hemangiomas consist of dilated capillary-like vascular spaces (see Figure 15) with bland endothelium and usually have one feeder vessel. Cavernous hemangiomas appear similar to markedly dilated vascular spaces that are thin-walled, venous vessels filled with blood [144]. There are reports of lesions being completely removed by CNB [144]. Cellular markers are rarely useful since markers will be similar to those in angiosarcoma [157].

Excision has been recommended for all benign vascular lesions to exclude angiosarcoma [150,155]; the rationale is twofold. First, if only well-differentiated areas are sampled, angiosarcomas may be underdiagnosed [144,155]. In newer retrospective case series, few cases where benign hemangiomas, even with atypia on CNB are upgraded to angiosarcoma [144,155]. For instance, in three case series (total *n* = 50) involving vascular breast lesions, only one was upgraded to a low-grade angiosarcoma after excision, and features were suspicious on pre-operative CNB [144,158,159]. There are older reports of up to 37% of angiosarcomas being missed on initial biopsy and called benign vascular lesions [160].

Second, there is some concern in the literature for malignant transformation which has been seen in extra-mammary hemangiomas and older, less certain reports from breast literature [161]. On this basis, surgery has been recommended for all hemangiomas in reviews as recently as 2022. Other reports suggest surgery could be avoided in patients with radio-pathologic concordance [144]. However, surveillance would be required; one report suggested surveillance every 6 months for 2 years, with no supporting studies quoted [162]. Excision would be recommended for any radio-pathologic discordance, interval growth, or high-risk pathologic findings [144,163]. Updated literature on upgrade rates and malignant transformation is required to make definitive recommendations.

#### Summary of Updates

A new classification system has been developed for vascular lesions by the ISSVA with most breast hemangiomas being benign proliferative lesions of capillaries by this classification. Management has remained unchanged over the years, with old literature supporting recent recommendations for surgical excision of all hemangiomas.

### 6.2. Atypical Vascular Lesion

Atypical vascular lesions are benign processes that occur in patients post-radiation [164]. They were first described by Finberg and Rosen in 1994 [165]. They were classified as their own entity by the World Health Organization in 2019 [164] and criteria were simultaneously established for diagnosis. These criteria include (1) previously irradiated skin, (2) dermal-based proliferation, rarely deep dermal, (3) relatively circumscribed, (4) irregularly shaped, thin-walled vascular spaces lined by a single layer of endothelial cells, (5) lack of infiltrative growth, cytological atypia and mitosis, (6) no MYC overexpression by IHC and (7) no MYC amplification by FISH [164].

After the definition by the WHO, a Danish study found a significant increase in AVL diagnoses, likely related to a defined consensus as well as the overall increase in breast-conservation surgeries and subsequent radiation exposure [164]. There are two subtypes (1) lymphatic and (2) vascular; however, these often overlap [28]. Compared to secondary angiosarcomas, AVLS are less common [28] and are present earlier, typically in the sixth decade of life [165]. They present as a singular or multiple purple or brown papule with a smaller average size than angiosarcomas, usually 5 mm or less [28,165], three to four years after radiation [165]. Ronen et al. describe a similar radiation-induced lesion presenting as hyperpigmented papules called benign lymphangiomatous papules (BLAP) that may represent another manifestation of AVLs [165]. Imaging is usually uninformative.

**Figure 16:**
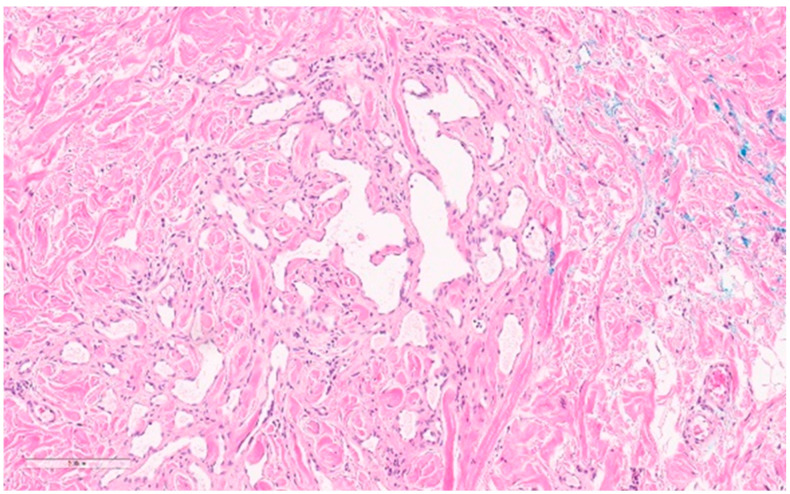
Atypical Vascular Lesion, Anastomosed lymphatic or capillary vessels, no mitoses or MYC rearrangements.

Microscopically, the lymphatic and vascular subtypes have anastomosing lymphatic or capillary vessels, respectively, located in the dermis (see Figure 16) [165] and lined by flat to hobnailed endothelial cells [28]. Hyperchromatic nuclei are often present [28]. Absent features differentiate these from secondary angiosarcomas and include nuclear multilayering, mitoses, prominent nucleoli, and atypia [28].

IHC staining that is positive for c-MYC has near 100% specificity for radiation-induced angiosarcoma and can be used to differentiate the two lesions [28,164]. However, this is only 80–90% sensitive and a negative test will not preclude a diagnosis of angiosarcoma [28]. Patton et al. classified AVLs into two groups, namely, lymphatic and vascular, differentiated by staining for D2–40 which was positive in the former [166]. They suggested vascular subtypes may have a higher risk of angiosarcoma development, but this has not been reproducible [167].

Given the risk of sampling error with biopsy diagnosis, it is often recommended that these be excised to a negative skin margin [28]. These lesions are reported to recur at rates of 10–20% [28]. They can also occur elsewhere in the same radiation field [168]. In a series of 11 Danish patients, 7 did not undergo excision and did not develop angiosarcoma in a median of 22 months follow-up [164]. Malignant transformation into angiosarcoma is rare. However, these patients carry a higher risk of angiosarcoma compared to the average population given the field defect created by radiation [168].

#### Summary of Updates

AVLs have recently had diagnostic criteria defined by the WHO. Molecular markers such as c-myc have shown some benefit in differentiating AVLs from angiosarcoma; D2–40 may help stratify the risk of malignant transformation, but this result has not been reproducible yet. With a proper definition, an aging population, and increased use of lumpectomy with radiation, AVLs are likely to continue increasing in incidence, which ideally will simultaneously increase our understanding.

## 7. Other

### 7.1. Pseudoangiomatous Stromal Hyperplasia

Pseudoangiomatous Stromal Hyperplasia (PASH) was first described by Vuitch, Erlandson, and Rosen in 1986 [169]. PASH is a stromal lesion, possibly originating from a benign proliferation of myofibroblasts, with the production of collagen that simulates a vascular lesion [169]. Though unusual as a solitary lesion, with fewer than 1500 cases described prior to 2020, it is a common incidental finding in association with other lesions [170,171].

Their etiology may be hormonal, a theory supported by their epidemiology. PASH occurs in women on oral contraceptives and hormone replacement, who are pregnant or peri-menopausal, or in men with gynaecomastia. In the largest series of male PASH (*n* = 44), only one did not have gynaecomastia [172]. One report suggested an association with psychiatric medications that increase levels of progesterone [173]. Histopathology findings also support this theory; high progesterone and variable estrogen receptor expression have been noted on spindle cells of PASH [173,174].

PASH tends to present in pre-menopausal women, though has been reported from 12 to 86 years old [175]. It has three forms: incidental (on biopsy or excision of another lesion) [176], nodular (mass-forming), or diffuse [177]. Incidental PASH has been found in 23% of breast biopsies and mastectomy specimens (*n* = 200) [171]. Their associations with other lesions were a cause of concern, with 11% of PASH noted to be associated with ductal carcinomas [178], and are now primarily thought to be coincidental [179]. However, a subsequent large retrospective review by Degnim et al. reported no increased risk of breast cancer after a biopsy of PASH. There have been case reports of giant mass-forming lesions [180]; in these unusual scenarios, they are often rapid-growing, painless lesions.

Given their ability to co-exist with other lesions, incidental PASH will be radiographically variable [180,181]. In nodular PASH, 22% are mammographically occult and the remainder present as a benign-appearing, hyperdense, circumscribed mass [176,178]. Reports vary on the incidence of calcifications [169,175]. Sonographically, nodular PASH will appear as a hypoechoic, oval, circumscribed mass with no posterior enhancement [169,175]. Some will have suspicious features including heterogeneity, increased echogeneity, or irregular borders [181]. Diffuse PASH will appear mammographically as enlarged dense breasts and sonographically as hypoechoic or heterogenous lesions with lace-like reticular hyperechoic areas and cysts [181].

In a series of 69 patients examining MRI findings for PASH, the most common presentation was clumped non-mass enhancement with persistent contrast kinetics, but overall, the MRI appearance varied significantly and the pathologic appearance and distribution of PASH varied within categories of MRI presentation [182]. In both nodular and diffuse PASH, heterogeneity with “slit-like” spaces has been described on T2-weighted images [177].

**Figure 17:**
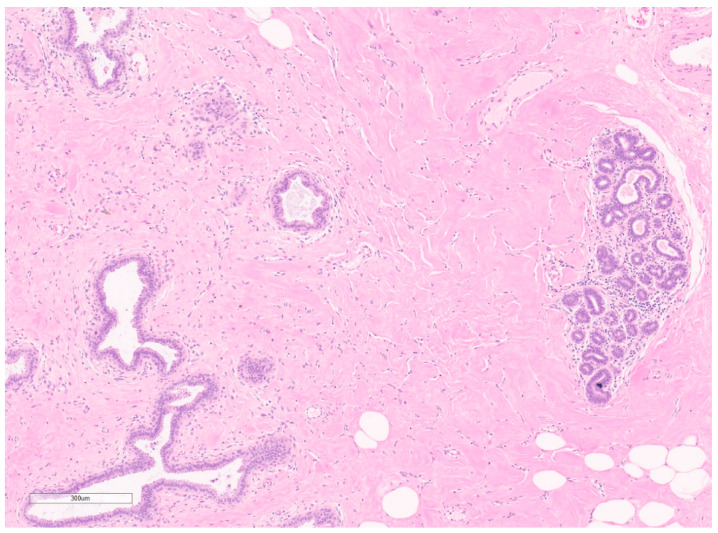
Pseudo-Angiomatous Stromal Hyperplasia, pseudo-vascular slit-like spaces without red blood cells.

Macroscopically, mass-forming PASH is a smooth, non-encapsulated, rubbery mass. Microscopically, for tissue diagnosis, FNA will often be acellular and is not useful [169,180]. One study showed confirmation of PASH on CNB in 63% of patients (*n* = 61) [181]. There is no evidence that CNB underestimated angiosarcoma for PASH [182]. They will appear as complex patterns of interanastomosing “slit-like” spaces lined with spindle cells (see Figure 17) [175], making them easily confused with blood vessels. PASH will have a lack of erythrocytes in true vascular spaces and a lack of nuclear atypia, mitoses, and pleomorphism compared to angiosarcoma. On IHC, they will stain positive for CD34, PR, and AR with variable desmin, vimentin, and SMA staining, supporting a myofibroblastic origin [175].

Surgical excision has historically been recommended in the literature (Kurt). The more recent 2016 American Society of Breast Surgery (ASBRS) guidelines and the 2018 American Society of Breast Surgeons Choosing Wisely Campaign recommend against excision [175,183]. Serial enlargement during observation, size exceeding 3 cm and radio-pathologic discordance remain indications of excision [175]. These indications have the primary aim of ruling out occult malignancy. In one series, 3/61 cases had pre-malignant lesions associated with PASH, and, in all 3, this was noticed on CNBs. There are no guidelines on required margins [175].

For patients being observed, serial mammography and clinical exams should be performed to assess growth [177]; intervals of 6 months have been recommended [177] though are poorly studied and recommendations could not be found in any guidelines [183]. Given that some reports suggest PASH patients develop breast cancers at lower rates than other women [175,179], they may not require surveillance beyond routine screening. Conservative management, per recent reports, can also include tamoxifen in symptomatic patients [175]. There is no evidence that PASH undergoes malignant transformation, but recurrences have been reported post-resection at rates of 7–22% [180].

#### 7.1.1. Summary of Updates

In the current era, with the accuracy of imaging and core needle biopsies, non-operative management has become the standard of care unless suspicious features are present. There remains a paucity of data on ideal surveillance.

#### 7.1.2. Summary Tables

Mesenchymal tumors are portrayed in this paper, excluding fibroepithelial lesions. These rare breast tumors have made varying degrees of progress in their diagnoses and management over the past 20 years. Desmoid tumors have made the most dramatic changes, with a non-operative approach taking precedence over previously recommended aggressive operative management. Others, such as hemangiomas, have seen little progress, and operative intervention is still recommended due to a poor understanding of natural history.

Below, we provide a summary of this paper in table format. Table 1 and Table 2 demonstrates an overview of fibroblastic/myofibroblastic tumors. This table also provides simplified methods of differentiating them from their common differentials. Table 3 demonstrates an overview of other tumor types.

## Figures and Tables

**Table 1:** Benign Fibroblastic/Myofibroblastic Tumor Summaries. Notes relating to differentiating common differential diagnoses for each tumor type are simplified and differentiation can be challenging.

Tumor Type	Presentation	Imaging Appearance	Biopsy	Pathology	Molecular Markers	Management	Prognosis
Nodular Fasciitis	Broad age range (20–80 years) Painless Rapid Growth Trauma 10–15%	US: hypoechoic, spiculated MG: hyperdense with indistinct margins	CNB may be sufficient with concordance and expert pathology Excisional biopsy usually needed	- Clusters of fibroblasts with short bundles - Myxoid bundles - Inflammatory cells (lymphocytes) - Mitotic figures	Positive: SMA FISH: + USP6 fusion gene (MYH9)	Excision—No research on required margins Increasing evidence to support observation of intra-lesional steroids (stemming from extra-mammary sources)	No metastases Low risk for recurrence
	Common Differentials and Features Differentiating them from NF: - Myofibroblastoma or Fibromatosis more heterogenous spindle cells, less organized - Metaplastic carcinoma: cytokeratin positivity - Sarcomas nuclear atypia (note: both will have mitotic figures) - Phyllodes tumors: large cohesive epithelial fragments/”fronds”						
Myofibro-blastoma	50–60 years old Male predominance Painless Slow-growing	US: well-circumscribed, homogenous, hyperechoic MG: well-circumscribed, coarse calcifications MRI: hyperintense on T2 and homogenous with septations	FNA insufficient CNB is usually sufficient Excisional biopsies rarely needed	- Many types: epithelioid, myxoid, deciduoid, schwannoma-like - Random arrangements of fascicular bipolar bland spindle cells - Interspersed adipocytes - Collagenous and myxoid background - Pseudocapsule	Positive: Desmin, CD34 (often co-expressed) Variably positive: CD10CD99, ER/PR, H-caldesmon Negative: S100, HMB45, EMA, pancyotkeratin Loss of RB1 expression (deletion on chromosome 13)	Local excision No studies on observation—natural history unknown	Two case reports of local recurrence No metastases
	Common Differentials and Features Differentiating them from Myofibroblastoma: - Fibromatosis: Negative CD34 - IMT Inflammatory infiltrates - Spindle cell lipoma Negative for desmin (note, will also have loss of RB 1 staining) - Cellular angiofibroma Usually not positive for both desmin and CD34, less fascicular arrangement of spindle cells (note, will also have loss of RB 1 staining) - Metaplastic carcinoma Positive keratins - Epithelial breast cancers (lobular) Positive keratins						
Fibromatosis	30–40 years old Painless Mobile Skin changes and nipple changes common	US: poorly defined, hypoechoic with echogenic rims and posterior shadows MG: high density, spiculated, rarely calcified MRI: report larger sizes than US/MG (should be the primary diagnostic method)	FNA insufficient CNB almost always sufficient Excisional biopsy only if review by expert pathologist is inconclusive	- Long spindle cells in interlacing fibroblastic bundles - “Tentacle-like” infiltrating margins - Bland cells, hyperchromatic nuclei, frequent nucleoli - Mitoses are rare - Lymphoid aggregates in periphery	Positive: Beta-katenin mutation SMA (Weak, focal) Variably positive: Desmin, S100, CD34 Negative: Cytokeratin, p63, ER/PR	Active surveillance: MRI (or CT) every 3–6months for 1–2 years. If progression is noted proceed with medical management. Tyrosine kinase inhibitors OR methotrexate with vinorelbine If ongoing progression, change medical management or consider radiation. Surgery is a last resort. Tumor will usually recur and surgery is deforming.	Variable depending on treatment regimen Recurrence extremely common with resection
	Common Differentials and Features Differentiating them from Fibromatosis: - Solitary fibrous tumor Positive CD34 (note: will stain positive for beta-catenin, making differentiation difficult) - Fibrosarcoma Nuclear polymorphism, high mitotic index, abnormal mitoses, necrosis, vascular invasion - Spindle cell carcinoma Positive cytokeratins (perform panel) - Fibrous histiocytoma Epithelioid cells, histiocytic cells or multinucleated cells						

**Table 2:** Borderline Malignant Fibroblastic/Myofibroblastic Tumor Summaries. Notes relating to differentiating common differential diagnoses for each tumor type are simplified and differentiation can be challenging.

Tumor Type	Presentation	Imaging Appearance	Biopsy	Pathology	Molecular Markers	Management	Prognosis
IMT	Young women Painless Slow-growing	US: lobulated, hypoechoic MG: hyperdense, calcifications may occur MRI: unclear margins, lobulated, rapid enhancement	CNB usually sufficient Excisional biopsy should be done with wide margins if diagnosis is suspected	- Variable proportions of spindle cells and stromal cells - Inflammatory infiltrate - Plump myofibroblasts - Mitoses but no atypia	Positive: SMA, MSA, vimentin, desmin Stain ALK1—positive in 50–60% can confirm diagnosis If ALK1 negative, ROS1/NTRK3 stain can confirm diagnosis Variably positive: cytokeratins Negative: p63, CD117, CD34, S100	Wide local excision with negative margins No evidence to support axillary surgery Extra-mammary cases have support from ALK inhibitors – consider in aggressive disease	Rare reports of metastases Up to 25% recurrence rates
	Common Differentials and Features Differentiating them from IMT: - Phyllodes and fibroadenomas: Epithelial “frond-like” growths, very similar on imaging - Myofibroblastoma Positive CD34 and CD10 - Nodular fasciitis Positive CD38 - Fibromatosis Beta-catenin expression - Spindle cell carcinoma Diffuse cytokeratin positive						
DFSP	20–30years old Slow-growing Painless Plaque/papule on the skin, centered in the dermis	US: parallel to the skin, plaque MG: hyperdense with no calcifications Note: can appear parenchymal on imaging	Biopsy: CNB or punch biopsy Excisional could also be considered	- Centered in the dermis or subcutis rather than the breast parenchyma - Hypercellular fascicles of spindle cells - Storiform architecture infiltrating - Cigar-shaped nuclei - Cartwheel pattern of nuclei - Fibrotic stroma	Positive: CD34, WT1 Negative: keratin, S100, SMA, desmin 90% have translocation t (17:22) from fusion of COL1A1 with PDFGB	Wide local excision No indication for axillary surgery Moh’s microsurgery is controversial and not useful in the breast Limited sensitivity to chemotherapy and radiation Imatinib can be considered (borderline resectable)	2–25% recurrence rates Metastasis occurs only with fibrosarcomatous progression (FS-DFSP)—occurs in 10–14%
	Common Differentials and Features Differentiating them from DFSP: - Metaplastic carcinoma Co-expression of AE1/AE3 and vimentin, in variants most similar to DFSP, CD34 is usually negative - Fibromatosis Desmin and SMA positive, CD34 negative - Phyllodes tumor Presence of epithelial and duct components should differentiate						

**Table 3:** Other Tumor Summaries.

Tumor Type	Presentation	Imaging Appearance	Pathology	Molecular Markers	Management	Prognosis
Leiomyoma	40–50 years old Slow-growing Painful—especially with cold and palpation	US: solid, homogenous, hypoechoic and well-circumscribed MG: homogenous dense lesions with well-defined margins and no calcifications MRI: well-circumscribed, whorl-appearing, low T1 intensity and low-intermediate T2 intensity with gradual enhancement	- Spindle cells in “cigar-shaped,” blunt ending nuclei - Eosinophilic cytoplasm	Positive: desmin, SMA, h-caldesmon Negative: CD34, p63, S100, keratins	Multiple leiomyomas: test for HLRCC syndrome Wide excision to negative margins (most described)	Natural history in the breast is poorly understood Considered similar to extra-mammary locations: indolent, non-aggressive No recurrences, malignant transformation
Neurofibroma	20–30 years old Male and female Often in NAC or pectoralis fascia Usually associated with NF1	US: superficial, circumscribed, hypoechoic nodules MG: hyperdense, well-circumscribed MRI: hypo- or iso- intense on T1 and hyperintesnse and heterogenous on T2	- Spindle cells with bland “comma-shaped” or serpentine nuclei - Smudgy chromatin - “Shredded-carrot-like” collagenous stroma	Positive: S100 and SOX10 Variably positive: CD34	Excision to negative margins Margins impact recurrences (for atypical lesions) Refer all for genetic counselling (assess for NF1)	Few local recurrences Note: NF1 patients have two times the lifetime increased risk of epithelial breast cancer
GCT	Painless, firm masses Upper inner quadrants ¼ identified through screening	US: hypoechoic with irregular borders MG: spiculated distortions without calcifications MRI: low T1 intensity, difficult to visualize on T2, variably enhancing PET: useful for differentiating benign GCTs from malignant	- Infiltrating sheets of polygonal bland cells - Well defined cell borders - Eosinophilic, granular cytoplasm - Stain with PAS (periodic acid Schiff)	Positive: S100, SOX10 and CD 68	Excision to negative margins	Local recurrences - 2–8% with negative margins - 20% with positive margins Surveillance recommended but no evidence for frequency and duration
Schwannoma	Rare in the breast Pain to palpation of a mass Associated with NF2	US: variable due to areas of degeneration or hemorrhage MG: variable, may be occult MRI: isointense on T1 and heterogenous on T2	- Varying degrees of encapsulation - Lymphoid cuffs - Clusters of hyalinized vessels - Alternating compact hypercellular (Antoni A) and myxoid hypocellular (Antoni B) regions - Nuclear palisading (Verocay bodies)	Positive: S100 and SOX10 suspect malignancy with loss of p16	Surgical enucleation is usually sufficient Extra-mammary evidence for gamma knife surgery	Local recurrences possible No metastases Rare malignant transformation
Leiomyoma	40–50 years old Slow-growing Painful—especially with cold and palpation	US: solid, homogenous, hypoechoic and well-circumscribed MG: homogenous dense lesions with well-defined margins and no calcifications MRI: well-circumscribed, whorl-appearing, low T1 intensity and low-intermediate T2 intensity with gradual enhancement	- Spindle cells in “cigar-shaped,” blunt ending nuclei - Eosinophilic cytoplasm	Positive: desmin, SMA, h-caldesmon Negative: CD34, p63, S100, keratins	Multiple leiomyomas: test for HLRCC syndrome Wide excision to negative margins (most described)	Natural history in the breast is poorly understood Considered similar to extra-mammary locations: indolent, non-aggressive No recurrences, malignant transformation
Neurofibroma	20–30 years old Male and female Often in NAC or pectoralis fascia Usually associated with NF1	US: superficial, circumscribed, hypoechoic nodules MG: hyperdense, well-circumscribed MRI: hypo- or iso- intense on T1 and hyperintesnse and heterogenous on T2	- Spindle cells with bland “comma-shaped” or serpentine nuclei - Smudgy chromatin - “Shredded-carrot-like” collagenous stroma	Positive: S100 and SOX10 Variably positive: CD34	Excision to negative margins Margins impact recurrences (for atypical lesions) Refer all for genetic counselling (assess for NF1)	Few local recurrences Note: NF1 patients have two times the lifetime increased risk of epithelial breast cancer
GCT	Painless, firm masses Upper inner quadrants ¼ identified through screening	US: hypoechoic with irregular borders MG: spiculated distortions without calcifications MRI: low T1 intensity, difficult to visualize on T2, variably enhancing PET: useful for differentiating benign GCTs from malignant	- Infiltrating sheets of polygonal bland cells - Well defined cell borders - Eosinophilic, granular cytoplasm - Stain with PAS (periodic acid Schiff)	Positive: S100, SOX10 and CD 68	Excision to negative margins	Local recurrences - 2–8% with negative margins - 20% with positive margins Surveillance recommended but no evidence for frequency and duration
Schwannoma	Rare in the breast Pain to palpation of a mass Associated with NF2	US: variable due to areas of degeneration or hemorrhage MG: variable, may be occult MRI: isointense on T1 and heterogenous on T2	- Varying degrees of encapsulation - Lymphoid cuffs - Clusters of hyalinized vessels - Alternating compact hypercellular (Antoni A) and myxoid hypocellular (Antoni B) regions - Nuclear palisading (Verocay bodies)	Positive: S100 and SOX10 suspect malignancy with loss of p16	Surgical enucleation is usually sufficient Extra-mammary evidence for gamma knife surgery	Local recurrences possible No metastases Rare malignant transformation
Lipoma	Most common tumor of human body 30–50years old Risk factors: obesity, dyslipidemia, diabetes Slow-growing masses	US: hypoechoic and avascular MG: radiolucent with a thin capsule with calcifications Imaging is indicated in mammary lipomas (not always necessary extra-mammary locations)	CNB indicated if imaging is not classic. Challenging due to adjacent adipose tissue Normal adipose tissue with small nuclei, interspersed septa Many histologic subtypes		Observation Weight loss may decrease size Injections of deoxycholate injections decreases size by 75% Removal to rule out liposarcoma (size, growth pattern, pain and fixation) The entire capsule should be removed if excising.	No metastases Recurrences may occur, if excision incomplete
Angiolipoma	50–70 years old More common in women Painless singular mass Often screen detected	US: well circumscribed, homogenous and hyperechoic MG: occult or circumscribed isodense, no calcifications MRI: low signal intensity on T1 and high signal intensity on T2	CNB is usually sufficient Mature adipose tissue Branching capillary vessels, clustered at the periphery Hyaline microthrombi	Positive: CD34, CD31, ER, S100, androgen receptor Focally positive: SMA	Excision not necessary with radio-pathologic concordance Undefined surveillance strategy Cellular subtype of angiolipoma—excision is indicated to rule out low grade angiosarcoma	No history of malignant transformation or recurrence
Hemangioma	50 years old Female predilection Seen in 11% of autopsies Superficial palpable masses with skin discoloration Often incidental on screening mammography	US: variable definitions, difficult to differentiate from complex cysts or fibroadenomas MG: non-specific, well-circumscribed, can have calcifications related to phleboliths MRI: circumscribed masses with fibrous septa, fast enhancement and washout	Expect bleeding with biopsy FNA inconclusive and hypocellular CNB usually needed Dilated vascular spaces with thin-walled venous vessels	Rarely useful—very similar to angiosarcoma	Excision indicated- to rule out angiosarcoma and for risk of malignant transformation Note: much of this comes from older literature. More studies are needed. If not excising, should have radio-pathologic concordance and should be on surveillance.	Low malignant transformation No metastases
Atypical Vascular Lesion	50 years old Must have history of radiation Single of multiple purple or brown papules Usually <5 mm	Usually uninformative	CNB or punch biopsy suggested Anastomosing lymphatic or capillary vessels in the dermis Flat to hobnailed endothelial cells Hyperchromatic nuclei	c-myc has 100% specificity but 80–90% sensitivity	Excise to negative margins	10–20% recurrence rates Malignant transformation is rare, but th field defect from radiation exposure confers a higher risk of angiosarcoma
Pseudo-angiomatous Stromal Hyperplasia	Pre-menopausal women Risk factors: hormonal exposure including contraceptives, pregnancy, hormone replacement, gynaecomastia	Incidental PASH: occult Nodular PASH: US: hypoechoic, well circumscribed MG: hyperdense, well circumscribed Diffuse: heterogenous, lace-like on US	FNA is often acellular and not useful CNB are adequate—no evidence that it underestimates angiosarcoma as PASH Complex inter-anastomosing channels with spindle cells No erythrocytes In true vascular spaces	Positive: CD34, PR and AR Variably positive: desmin, vimentin, SMA	Most lesions can be observed (no clear surveillance regimen) Surgical excision for PASH > 3 cm, radio-pathologic discordance or serial enlargement No consensus on margins	No malignant transformation Recurrence rates 9–22%
